# Intraspinal Azygos Vein

**DOI:** 10.5334/jbsr.2911

**Published:** 2022-11-03

**Authors:** Joanna Knapik, Elke Vereecke, Lieve Morbée

**Affiliations:** 1 UZ Gent, BE

**Keywords:** intraspinal, azygos vein, anatomical variant, magnetic resonance imaging

## Abstract

**Teaching Point:** Intraspinal azygos vein is an extremely rare anatomical variant; knowledge is important for correct imaging interpretation.

## Case History

**Figure 1:**
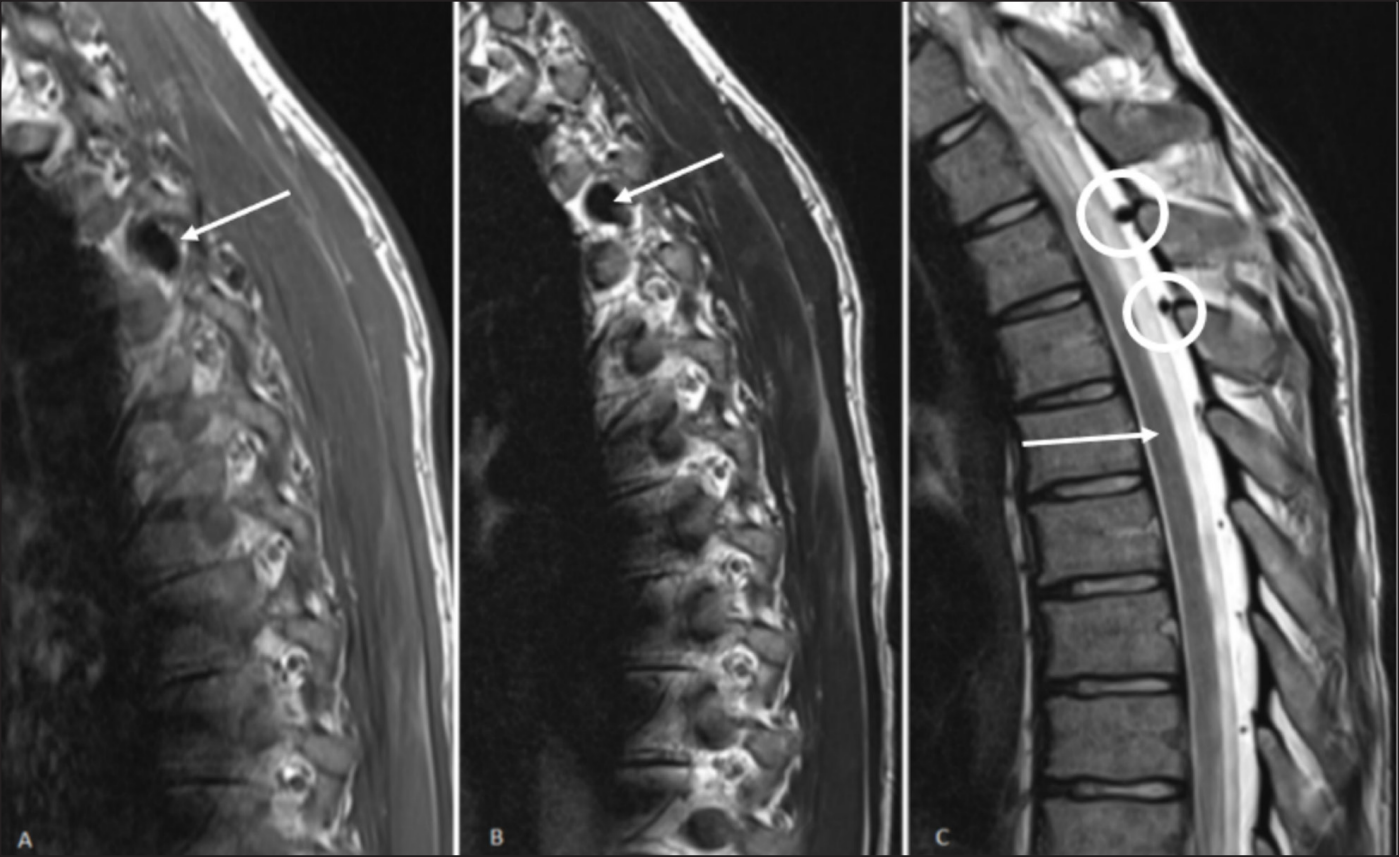


A 26-year-old man was referred to our radiology department with atypical complaints of muscle tension and upper back pain localized mainly midthoracic and radiating anteriorly. Given his young age, the patient initially underwent magnetic resonance imaging (MRI) of the thoracal and lumbar spine. No relevant abnormalities of the thoracic vertebra, intervertebral discs, facet joints or spinal cord were present. However, a nodular structure in the right neural foramen Th3-Th4 was detected. This structure was hypointense on T1- (Figure 1A, sagittal view, arrow) and T2-weighted turbo spin echo images (Figure 1B and 1C, sagittal view, arrows). The structure was connected to several enlarged posterior epidural vessels (Figure 1C, sagittal view, circles). Assumption of an aberrant vascular structure was made.

**Figure 2:**
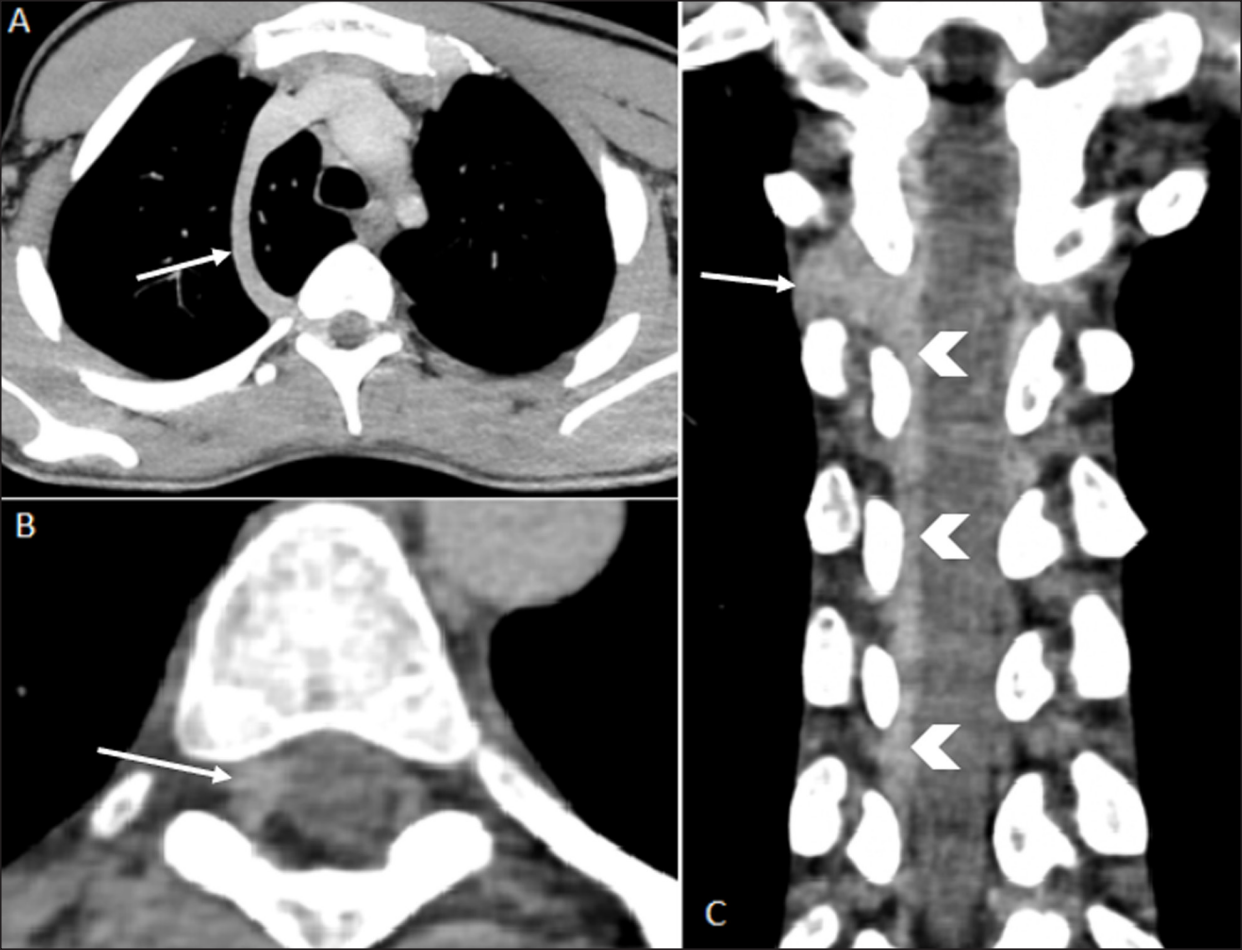


**Figure 3:**
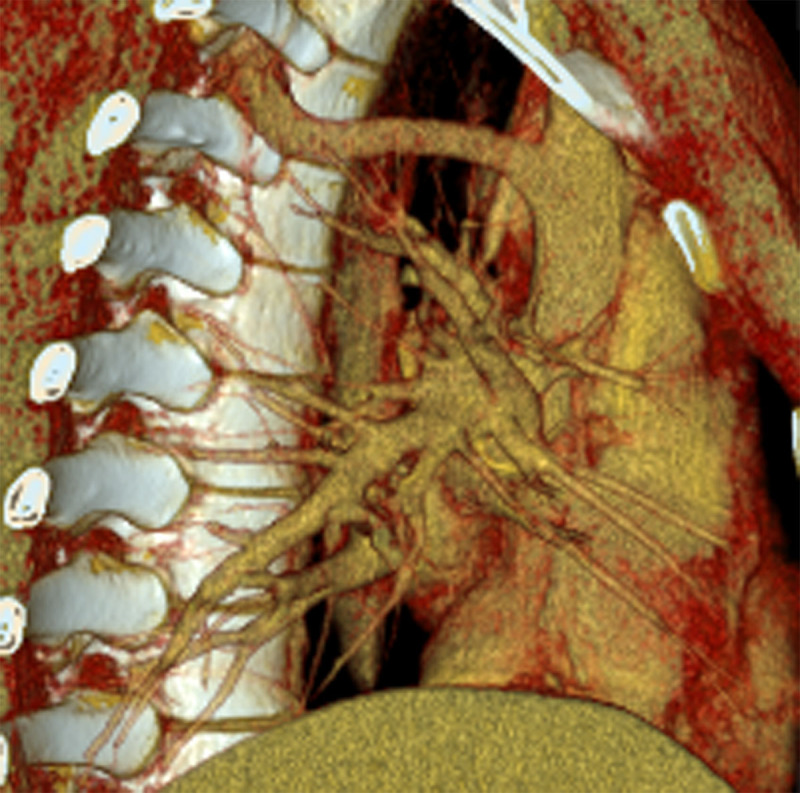


To better delineate these vascular structures and to exclude associated intrathoracic abnormalities, an additional computed tomography (CT) imaging was performed with injection of intravenous contrast material (both venous and arterial phase were acquired). This demonstrated an azygos lobe in the right lung (Figure 2A, semi-axial MIP, arrows). Visualized azygos vein passed further through the right Th3-Th4 neural foramen (Figure 2B, semi-axial MIP, Figure 2C, semi-coronal reformation, arrows and Figure 3, 3D reformatting) and ran caudally intraspinal in the right extradural space to level Th12 (Figure 2C, arrow heads). No additional intrathoracic vascular abnormalities were detected.

Based on the new data, a diagnosis of an intraspinal azygos vein was made. An interventional radiologist was consulted, yet invasive treatment was not recommended. Back pain and muscle tension were treated non-invasively with analgesics and physiotherapy.

## Comment

Azygos system consists of azygos, hemiazygos and accessory hemiazygos veins. It provides collateral circulation between superior and inferior vena cava. Azygos vein originates as a union of the right ascending lumbar vein and right subcostal veins (Th12-L2) and enters the chest through the aortic hiatus. Therefore, azygos vein ascends in the posterior mediastinum and arches over the right main bronchus to join the superior vena cava. Multiple anatomical variants exist, including azygos lobe, azygos continuation of the inferior vena cava or absence of the azygos vein. Intraspinal azygos vein is an extremely rare variant and according to our knowledge there is one other case reported in the literature [1]. There is no direct link between upper back pain and aberrant course of azygos vein. However, knowledge of described variant is important for correct interpretation of medical imaging and for performing surgeries or endovascular procedures.
